# Chlorogenic Acid Alleviates the Detrimental Effects of Concurrent Hyperglycemia and Chronic Stress on Brain Homeostasis by Modulating Antioxidative Defense in Adult Zebrafish

**DOI:** 10.3390/antiox14121386

**Published:** 2025-11-21

**Authors:** Rhea Subba, Gianluca Fasciolo, Adriana Petito, Eugenio Geremia, Maria Teresa Muscari Tomajoli, Amal Chandra Mondal, Gaetana Napolitano, Paola Venditti

**Affiliations:** 1 Laboratory of Cellular and Molecular Neurobiology, School of Life Sciences, Jawaharlal Nehru University, New Delhi 110067, India; rhea31_sls@jnu.ac.in; 2 Department of Biology, University of Naples Federico II, 80143 Naples, Italy; gianluca.fasciolo@unina.it (G.F.); adriana.petito@unina.it (A.P.); venditti@unina.it (P.V.); 3 International PhD Programme, UNESCO Chair “Environment, Resources and Sustainable Development”, Department of Science and Technology, Parthenope University of Naples, 80143 Naples, Italy; eugenio.geremia@studenti.uniparthenope.it (E.G.); mariateresa.muscaritomajoli001@studenti.uniparthenope.it (M.T.M.T.)

**Keywords:** astroglia, chlorogenic acid, diabetes distress, hyperglycemia, oxidative stress, zebrafish

## Abstract

Oxidative stress is a key contributor to diabetes-related cognitive decline and is intensified by diabetes distress (DD), the psychological burden of disease management. DD lowers brain levels of nuclear factor erythroid 2-related factor 2 (NRF2), a transcription factor that regulates antioxidant defense. This study examined whether chlorogenic acid (CGA), a polyphenolic NRF2 activator, could counteract oxidative and astroglial dysfunctions in adult zebrafish subjected to chronic unpredictable mild stress (CUMS) combined with dextrose, a model mimicking DD. Zebrafish were treated with CGA (50, 100, or 200 mg/kg), and the levels of NRF2 protein and mRNA, along with its regulator *keap1*, were quantified. Expression levels of key downstream antioxidant genes (*sod1*, *sod2*, *catalase*, *glutathione peroxidase*, and *glutamate-cysteine ligase catalytic subunit*) were assessed alongside glutathione (GSH) content and superoxide dismutase (SOD) and catalase activities. Astroglial integrity was evaluated via glial fibrillary acidic protein (GFAP) levels in the whole brain and stress-sensitive regions. CGA increased total brain NRF2 protein, its mRNA, and those of its downstream effectors. At 200 mg/kg, CGA restored GSH levels, boosted antioxidant enzyme activities, and mitigated DD-associated reductions in GFAP and NRF2 in stress-vulnerable areas. These findings identify NRF2 as a promising target to protect brain health under diabetic conditions.

## 1. Introduction

Diabetes mellitus, a chronic metabolic disorder, currently affects 589 million individuals worldwide, a number predicted to increase to 853 million by 2050 [1]. In the central nervous system (CNS), chronic hyperglycemia contributes to cognitive decline through mechanisms including oxidative stress, inflammation, insulin resistance, synaptic dysfunction, blood–brain barrier impairment, and neuronal death [2].

An often-overlooked contributor to disease burden is diabetes distress (DD), the psychological strain associated with diabetes management, which encompasses emotional, social, and treatment-related challenges [3]. DD considerably impairs glycemic control, medication adherence, and self-care practices, thereby worsening clinical outcomes. Although widely recognized as a critical barrier to effective diabetes management, the molecular mechanisms through which DD contributes to disease pathogenesis remain inadequately characterized.

A growing body of evidence highlights a reciprocal relationship between hyperglycemia and oxidative stress, which forms a self-reinforcing cycle that exacerbates disease progression [4]. Therefore, interventions targeting oxidative stress may offer therapeutic potential in mitigating diabetes-related complications [5]. One of the central regulators of cellular antioxidant defense is nuclear factor erythroid 2-related factor 2 (NRF2), a transcription factor that orchestrates the expression of antioxidant response element (ARE)-driven genes that are involved in detoxification, redox homeostasis, and cytoprotection. Under basal conditions, NRF2 is sequestered in the cytoplasm by its endogenous repressor Kelch-like ECH-associated protein 1 (KEAP1), resulting in its ubiquitination and proteasomal degradation via the Keap1/CUL3/RBX1 E3 ubiquitin ligase complex [6]. In response to oxidative or electrophilic stress, NRF2 dissociates from KEAP1, translocates to the nucleus, and dimerizes with small Maf proteins to initiate the transcription of target genes.

Previous studies in diabetic rodents have demonstrated brain-region-specific reductions in NRF2 protein levels, including in the hippocampus [7]. Consistent with these findings, our prior study on a zebrafish model of DD that was induced using chronic unpredictable mild stress (CUMS) combined with dextrose exposure, revealed redox imbalance along with reduced NRF2 levels in the whole brain [8]. Given the strong association between diminished NRF2 signaling and the pathogenesis of chin a zebrafish model of DD induced by chronic unpredictable mild stress (CUMS) combined with dextrose exposure revealed redox imbalance andronic diseases, including diabetes, pharmacological activators of NRF2 are under investigation for their potential therapeutic utility [9]. Among these, chlorogenic acid (CGA), a plant-derived polyphenol, has emerged as a promising candidate. CGA exerts potent antioxidant effects by directly scavenging reactive oxygen species (ROS) and activating NRF2 signaling to promote the transcription of antioxidant enzymes [10]. Additionally, CGA has shown efficacy in models of both chronic stress [11] and hyperglycemia [12], supporting its potential role in alleviating diabetes distress.

Beyond neuronal dysfunction, glial pathology, particularly involving astroglia, plays a critical role in CNS disorders. Astroglia are essential for neurotransmission, synaptic maintenance, antioxidant defense, and neuroprotection [13]. Astroglial dysfunction may manifest either as a gain of toxic function (reactive astrogliosis) or loss of homeostatic function (atrophy), depending on the disease context [13]. Glial fibrillary acidic protein (GFAP), a structural protein and marker of astroglial integrity, is differentially expressed in various pathologies: elevated in neuroinflammatory conditions, including Alzheimer’s disease and epilepsy, and attenuated in neuropsychiatric disorders such as major depressive disorder [14]. In rodents exposed to chronic stress, reduced GFAP expression and signs of astrocyte atrophy have been observed in the hippocampus and prefrontal cortex [15,16]. Conversely, diabetic conditions are associated with astroglial reactivity, neuroinflammation, and cognitive dysfunction [17].

Given the diverse manifestations of glial pathology in response to different stressors, targeted therapeutic strategies require a nuanced understanding of glial responses in specific disease contexts. Despite increasing recognition of glial contributions to CNS pathology, the role of astroglial dysfunction in the setting of diabetic distress remains unclear.

The present study aimed to determine whether CGA treatment could enhance antioxidant defense by attenuating CUMS+D-induced disturbances in brain NRF2 levels and its downstream effectors. Moreover, we investigated the impact of CUMS+D on GFAP-positive glial cells and evaluated the potential of CGA to modulate glial integrity.

## 2. Materials and Methods

### 2.1. Animals

Adult zebrafish were obtained from the Department of Biology farm at Federico II University of Naples. They were fed brine shrimp flakes twice daily (Ocean Nutrition Brine Shrimp Plus Flakes, Leland, NC, USA) and maintained in a temperature-regulated environment (26–28 °C) with a 14 h light/10 h dark photoperiod cycle. They were acclimated for at least two weeks before the experiments. Mature zebrafish, over six months old and measuring 2.0 ± 1.0 cm in length, were selected for the study.

The animal experimentation described in this study complied with the EU Directive 2010/63 on the care and use of laboratory animals. The study was authorized by the Scientific Ethics Committee of the Federico II University of Naples and the Italian Ministry of Health (Ministerial Authorization no. 767/2023-PR on 30 August 2023).

#### 2.1.1. Experimental Groups

**Figure 1:**
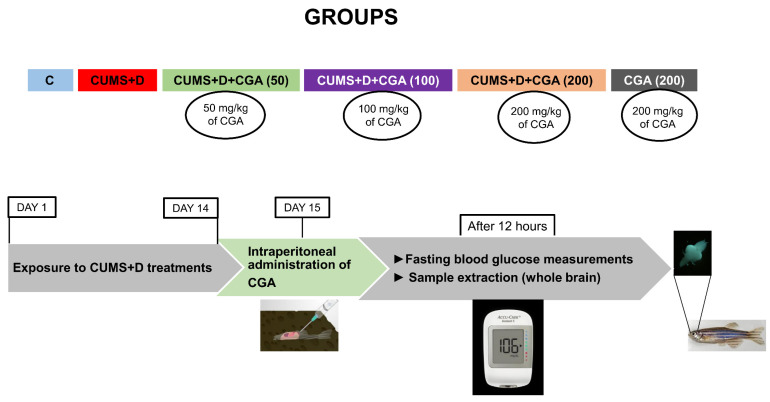
Experimental design and timeline of the study. Fish were allocated into six groups: Control (C), CUMS+Dextrose (CUMS+D), CUMS+Dextrose+CGA (CUMS+D+CGA 50, 100 or 200 mg/kg), and CGA (200 mg/kg). The fish underwent 14 days of CUMS+D treatment, followed by intraperitoneal CGA administration on Day 15. Fasting blood glucose was measured on Day 16, followed by extraction of whole-brain samples.

A total of 288 zebrafish were used in the study. Both male and female fish were included, and only healthy fish were used for the experiments. Fish were randomly assigned to six groups: a control group, Control; a group subjected to combined CUMS and Dextrose treatments, CUMS+D; a group subjected to combined CUMS and Dextrose treatments followed by an acute dose of 50 mg/kg CGA, CUMS+D+CGA (50); a group subjected to combined CUMS and Dextrose treatments followed by an acute dose of 100 mg/kg CGA, CUMS+D+CGA (100); a group subjected to combined CUMS and Dextrose treatments followed by an acute dose of 200 mg/kg CGA, CUMS+D+CGA (200); a control group administered an acute dose of 200 mg/kg CGA, CGA (200). The fish were allocated to different tanks, each labeled for a specific group. Initially, a pilot dose–response study was performed to identify the optimal dose and treatment duration of CGA treatment; fish were injected with 50, 100, 200, or 400 mg/kg per body weight doses of CGA. This resulted in only 50% survival at the 400 mg/kg dose (Supplementary Figure S1). Fish that received CGA doses at 50, 100, or 200 mg/kg displayed more than 80% survivability 24 h post-injection; therefore, these three doses were selected for the full-fledged experiments. Due to high mortality caused by recurring CGA injections, acute CGA doses (50, 100, or 200 mg/kg) were used in the final study, where fish in the experimental groups were exposed to CUMS+D for 14 days, followed by an acute dose of CGA on the 15th day. The experiment timeline and design are depicted in Figure 1.

The 288 fish used in this study were distributed as follows: Immunofluorescence: 3 fish per group × 6 groups = 18 fish; Western blotting: 3 pools of 5 brains per group × 6 groups = 90 fish; Biochemical assays and qRT-PCR: same sampling as for Western blotting (90 + 90) = 180 fish.

#### 2.1.2. Concurrent Exposure to CUMS+D

To induce chronic hyperglycemia, fish were exposed to a dextrose (Sisco Research Laboratories Pvt. Ltd., Mumbai, India, catalog no. 42738) solution (111 mM) following the methodology of an established protocol [18]. The dextrose solution was replaced every 24 h throughout the treatment to maintain a constant dextrose concentration and prevent microbial growth. Fish were simultaneously subjected to CUMS according to the protocol of a previous study [8]. The procedure involved the utilization of seven stressors in total: elevation, dorsal body exposure, chasing, tank changes, restraint, overcrowding, and isolation. These stressors are designed to emulate the chronic and unpredictable stressors that contribute to the development of stress-related neuropsychiatric conditions in humans, where cognitive dysfunction is a central feature [19]. Psychosocial stress associated with mental health disorders is also highly prevalent among individuals with chronic diseases, including metabolic disorders [20]. The parallels between these stressors and the CUMS stressors employed in this study can be described as follows: (a) elevation stress, which involves removing the fish from its natural aquatic environment using a net, primarily evokes a state of physiological stress accompanied by helplessness and panic; (b) dorsal body exposure, achieved by lowering the tank water level to expose the dorsal surface of the fish to air, similarly induces physiological stress and a partial state of immobility; (c) chasing with a net provokes fear, perceived threat, panic, and anxiety, eliciting both physiological and psychological stress associated with the fight-or-flight response; (d) tank changes, involving transfer from the home tank to a novel environment, trigger anxiety, fear, and novelty-related stress that mimic the psychological effects of environmental instability; (e) restraint in a 2 mL microcentrifuge tube effectively induces immobilization stress (a state of helplessness and loss of agency) representing a combination of psychological and physiological stress; (f) overcrowding, created by placing fish in a small beaker containing only 200 mL of water, leads to social crowding and resource competition, encompassing a psychosocial stress component; and (g) isolation in individual beakers effectively models social deprivation and loneliness, capturing another key dimension of psychosocial stress. To avoid habituation and ensure effective stress induction, these stressors were applied at random and in arbitrary order. Fish were exposed to two distinct stressors delivered unpredictably, with the nature of the stressors varying daily. The timing of stressor exposure was not fixed; the fish were subjected to stressors at random intervals throughout the day. Stressors were presented either in close succession or with longer intervals between exposures. The combined CUMS+D exposure lasted for 14 days.

#### 2.1.3. CGA Treatment

CGA (Sigma Aldrich, St. Louis, MO, USA, catalog number C3878) was dissolved in 10% dimethyl sulfoxide (DMSO, Sisco Research Laboratories Pvt. Ltd., Mumbai, India, catalog no. 28580), and vehicle control groups received an equivalent volume of 10% DMSO. Minor adjustments were made to the intraperitoneal injection protocol [21]. The fish were first anaesthetized using the hypothermia method. Each fish was placed in 100 mL of water, and ice chips were gradually added to lower the water temperature to approximately 10 °C. Anesthesia induction was confirmed by observing partial loss of equilibrium, reduced swimming, decreased respiratory rate, slowed operculum movements, and lack of response to tactile stimuli. Each fish was then briefly dabbed with absorbent tissue paper, following which body weights were recorded using a weighing balance. Based on body weight, 1–1.5 µL of CGA was injected intraperitoneally using a 26-gauge Hamilton syringe (Hamilton Company, Bonaduz, Switzerland). Following the injection, the fish were returned to regular aquarium water for recovery.

### 2.2. Fasting Blood Glucose Level Measurement and Brain Sample Extraction

Twelve hours after completing the CUMS+D exposure (Days 1–14) and CGA administration (Day 15), fasting blood glucose level measurements of 15 fish in each group were performed, followed by euthanasia for brain harvesting. The fish were first rinsed in dextrose-free water to remove any remaining dextrose from the external body surface. Euthanasia involved two steps: anesthesia, followed by hypothermic shock at 0–4 °C for 5 min [22]. Euthanasia was confirmed by the absence of bodily and operculum movements, lack of response to all stimuli, and ultimately by decapitation. Blood glucose levels were then recorded using a glucometer (Accu-Chek^®^ Instant S Blood Glucose Monitor, ACCU-CHEK^®^, Roche Diabetes Care GmbH, Sandhofer Straße, Mannheim, Germany). Whole brain samples were harvested and prepared for further experimental analyses.

### 2.3. Evaluation of Oxidative Stress

Whole brain samples were homogenized in phosphate saline buffer (PBS) (0.1 M at pH 7.4) to obtain a 10% homogenate. The parameters assessed were: (i) superoxide dismutase (SOD) and catalase enzyme activities, and (ii) glutathione (GSH) levels. For each assay, three biological replicates (*n* = 3) were used, with five whole brains pooled per replicate. Each data point corresponds to the mean of three technical replicates. Bradford assay [23] was performed to determine protein content in the samples.

#### 2.3.1. SOD Activity

SOD activity was assessed by its capacity to inhibit pyrogallol (Sigma Aldrich, St. Louis, MO, USA, catalog no. P0381) autoxidation [24]. Initially, 15 µL of the sample was added to 3 µL of 12.5% Triton X-100 (Sisco Research Laboratories Pvt. Ltd., Mumbai, India, catalog no. 64518) and incubated for 30 min at 4 °C. The final assay mixture consisted of 90 µL of pyrogallol solution (10.2 mg pyrogallol + 10 mL double-distilled water + 8.6 µL HCl), 49.5 µL of 3 mM EDTA (Sisco Research Laboratories Pvt. Ltd., Mumbai, India, catalog no. 43272) (pH 8), 750 µL of 0.1 M PBS, and 595.5 µL of double-distilled water. Absorbance was measured kinetically at 420 nm. Using the molar extinction coefficient (ɛ) of 800 × 10^3^ M^−1^ cm^−1^, SOD activity was calculated as µ moles of pyrogallol protected from oxidation per min per mg of protein.

#### 2.3.2. Catalase Activity

Catalase activity was determined by measuring the rate of dissociation of hydrogen peroxide (H_2_O_2_) (Qualigens, New Delhi, India, catalog no. Q18755) using a standard protocol [25]. A mixture of 500 µL of 0.05 M H_2_O_2_ and 975 µL of 0.1 M PBS (pH 7.4) was combined with 25 μL of the sample. Absorbance was measured kinetically at 240 nm. The molar extinction coefficient of 39.6 M^−1^ cm^−1^ was used to quantify catalase activity. The results were reported as µmoles of H_2_O_2_ consumed per min per mg of protein.

#### 2.3.3. GSH Content

Ellman’s reagent or 5,5′-Dithiobis (2-nitrobenzoic acid) (DTNB, Sigma Aldrich, St. Louis, MO, USA, catalog no. D8130) was used for the colorimetric measurement of total acid-soluble sulfhydryl concentrations to estimate GSH levels [26]. Initially, 150 µL of the sample was added to 150 µL of 4% sulphosalicylic acid solution, incubated for 1 h at 4 °C, and then centrifuged at 4000 rpm for 15 min. Thereafter, 200 μL of the supernatant was combined with 200 µL of DTNB (10 mM) and 1.1 mL of 0.1 M PBS (pH 7.4). Absorbance was recorded at 412 nm. Using the molar extinction coefficient of 1.36 × 10^4^ M^−1^ cm^−1^, GSH content was calculated and expressed as µ moles of GSH per g of tissue.

### 2.4. Expression of Oxidative Stress-Sensitive Markers

#### 2.4.1. Western Blotting

Immunoblotting was performed in biological triplicate (*n* = 3), with five whole brains pooled per replicate. Brain tissue (30 mg) was homogenized in 300 µL RIPA buffer with antiprotease cocktail. Protein lysates (60 µg) were separated on a 10% SDS-PAGE gel and transferred to a 0.2 µM PVDF membrane (Bio-Rad, Hercules, CA, USA, catalog no. 1620177). Membranes were blocked with 5% bovine serum albumin (BSA) (Sisco Research Laboratories Pvt. Ltd., Mumbai, India, catalog no. 85171) for 1 h at room temperature, and then incubated overnight at 4 °C with primary antibodies: (a) rabbit polyclonal anti-NRF2 primary antibody (1:500 dilution, Invitrogen, ThermoFisher Scientific, Waltham, MA, USA, catalog no. PA5-27882), (b) rabbit polyclonal anti-GFAP primary antibody (1:1000 dilution, GTX128741, GeneTex, Irvine, CA, USA, catalog no. GTX128741), and (c) rabbit polyclonal anti-β-actin primary antibody (1:2500 dilution, Abcam, Cambridge, UK, catalog no. ab8227); all primary antibodies were diluted in 5% BSA. Membranes were then washed three times for 10 min each with tris-buffered saline containing 0.1% of Tween-20 (TBST) and incubated for 1 h with an anti-rabbit HRP-conjugated secondary antibody (1:2000 dilution, Cell Signaling Technology, Danvers, MA, USA, catalog no. 7074S) that was diluted in 1% BSA. Another series of washing steps followed this. Protein bands were developed on X-ray films using the enhanced chemiluminescent technique (Clarity Western ECL Substrate, Bio-Rad, Hercules, CA, USA, catalog no. 1705061). Densitometric analysis was conducted using ImageJ software (version 1.53a), with beta-actin (β-actin) as the internal loading control.

#### 2.4.2. Immunostaining

Immunostaining was performed on coronal sections of the telencephalon using three biological replicates (*n* = 3), following an established protocol [27]. Fish heads were fixed overnight in 10% formalin at 4 °C. Intact brains were extracted and then fixed in 100% methanol (Sisco Research Laboratories Pvt. Ltd., Mumbai, India, catalog no. 96446) at −20 °C for at least 16 h. Brains were rehydrated through a series of methanol dilutions (75%, 50%, and 25%) and subsequently embedded in 2% agarose (Sisco Research Laboratories Pvt. Ltd., Mumbai, India, catalog no. 36601). Free-floating sections, 30 µm thick, were prepared using a vibratome (Leica VT-1200S, Leica Biosystems, Nussloch, Germany). The sections were initially incubated in a blocking solution (0.2% BSA) (Sisco Research Laboratories Pvt. Ltd., Mumbai, India, catalog no. 85171) for 1 h at room temperature, and then overnight at 4 °C with primary antibodies: rabbit polyclonal anti-NRF2 primary antibody (1:500 dilution, Invitrogen, ThermoFisher Scientific, Waltham, MA, USA, catalog no. PA5-27882) and rabbit polyclonal anti-GFAP primary antibody (1:1000 dilution, GTX128741, GeneTex, Irvine, CA, USA, catalog no. GTX128741). Following this, the sections were washed and incubated with goat anti-rabbit Alexa fluor^®^-488-conjugated secondary antibody (1:1000 dilution, Abcam, Cambridge, UK, catalog no. ab150077) for 2 h at room temperature. For counterstaining, 300 nM of 4′,6-diamidino-2-phenylindole (DAPI) (Sisco Research Laboratories Pvt. Ltd., Mumbai, India, catalog no. 18668) was used. Sections were mounted using fluoromount aqueous mounting solution (Fluoromount-G^®^, Southern Biotech, Birmingham, AL, USA, catalog no. 0100-01) and imaged using a fluorescent microscope (Eclipse Ti-E, Nikon, Tokyo, Japan) at 10× magnification and a confocal microscope (Nikon A1R confocal, Tokyo, Japan) at 10× and 60× magnifications.

#### 2.4.3. Quantitative Real-Time Polymerase Chain Reaction (qRT-PCR)

Total RNA was extracted from homogenized brain tissue using the GeneJET RNA purification kit (Thermo Scientific™, Vilnius, Lithuania, catalog no. K0731) according to the manufacturer’s instructions. Five whole brains were pooled for each biological replicate, resulting in a total of three biological replicates (*n* = 3). RNA concentration was measured using a Nanodrop 2000 Spectrophotometer (Thermo Scientific™, Waltham, MA, USA). The Verso cDNA synthesis kit (Thermo Scientific™, Waltham, MA, USA, catalog no. AB1453A) was utilized to convert 20 ng of total RNA to cDNA. qRT-PCR was performed on a CFX96 detection system (Bio-Rad, Hercules, CA, USA) using the PowerUp^TM^ SYBR^TM^ Green master mix (Applied Biosystems^TM^, Vilnius, Lithuania, catalog no. A25742). The cycling protocol began with an initial heating step of 2 min at 95 °C. This was followed by 40 cycles, each consisting of a denaturation phase at 95 °C for 5 s and an annealing phase at 60 °C for 30 s. A dissociation curve analysis was conducted to verify the specificity of the reaction. Table 1 lists the primer sequences for the evaluated genes. Relative mRNA expression levels of the target genes were determined using the 2^−ΔΔCT^ technique. *β-actin* was used as the endogenous control to normalize the mRNA fold change across the experimental groups.

### 2.5. Statistical Analysis

Quantified data are presented as mean ± standard error of the mean (SEM). Statistical analysis was performed using GraphPad Prism software version 8. Differences between experimental groups were assessed using a one-way analysis of variance (ANOVA), followed by Dunnett’s multiple comparisons test. Statistical significance was denoted as follows: * *p* < 0.05, ** *p* < 0.01, *** *p* < 0.001, and **** *p* < 0.0001 for comparisons with the control group, and ^#^ *p* < 0.05, ^##^ *p* < 0.01, ^###^ *p* < 0.001, and ^####^ *p* < 0.0001 for comparisons with the CUMS+D group.

## 3. Results

### 3.1. CGA Treatment Reversed CUMS+D Exposure-Induced Systemic Hyperglycemia

**Figure 2:**
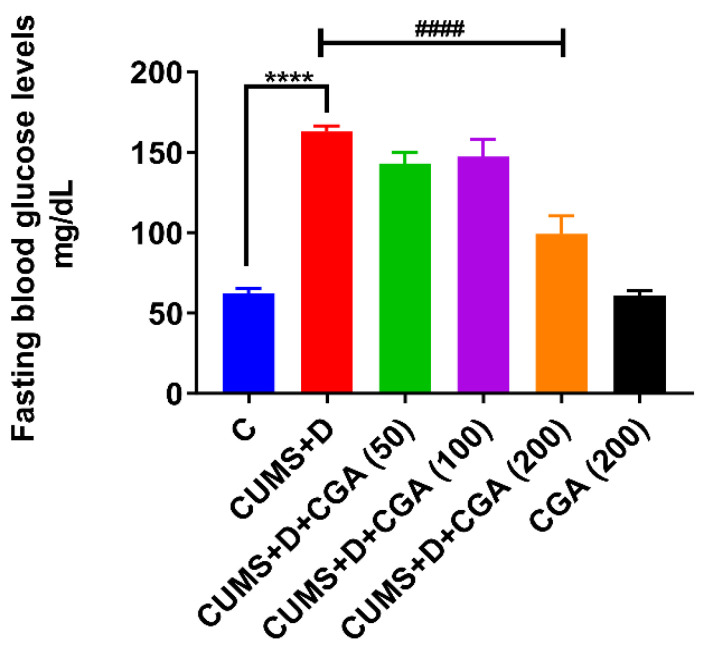
Effect of chlorogenic acid (CGA) treatment on fasting blood glucose levels in chronic unpredictable mild stress and dextrose (CUMS+D)-exposed zebrafish. All original data are reported in Supplementary Material S3. Significant differences are indicated by **** *p* < 0.0001 when compared with the control group (C) and ^####^ *p* < 0.0001 compared with the CUMS+D group. Data are presented as mean ± SEM (*n* = 15). Values represent the mean ± SEM (*n* = 15) from independent fish.

Fasting blood glucose levels (Figure 2) were significantly higher in the CUMS+D group than in the control group. CGA administration at a dose of 200 mg/kg significantly counteracted this elevation, nearly restoring fasting blood glucose to normoglycemic levels. Additionally, blood glucose levels in the CGA control group that were solely treated with 200 mg/kg of CGA were not significantly different from those in the control group.

### 3.2. CGA Treatment Modulated Antioxidative Defense in the Brains of Fish Exposed to CUMS+D

**Figure 3:**
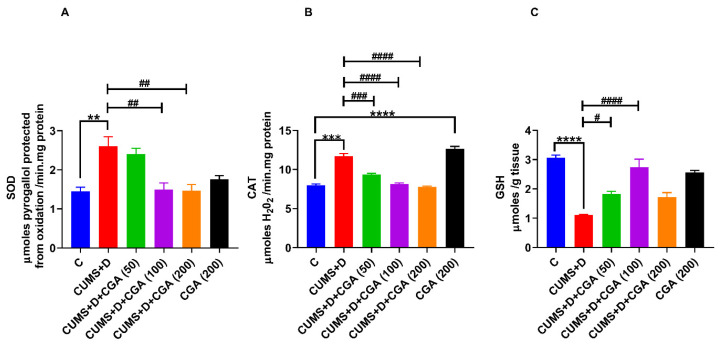
Effect of chlorogenic acid (CGA) treatment on antioxidant enzymes and glutathione (GSH) content in the whole brains of chronic unpredictable mild stress and dextrose (CUMS+D)-exposed zebrafish. (**A**) Superoxide dismutase (SOD) activity, (**B**) catalase activity, and (**C**) GSH levels. All original data are reported in Supplementary Material S3. Significant differences are indicated by **** *p* < 0.0001, *** *p* < 0.001, and ** *p* < 0.01 when compared with the control group (C); and ^####^ *p* <0.0001, ^###^ *p* <0.001, ^##^ *p* <0.01, and ^#^ *p* < 0.05 compared with the CUMS+D group, analyzed by one-way ANOVA followed by Dunnett’s multiple comparisons test. Values represent the mean ± SEM (*n* = 3) from three independent samples, each consisting of a pool of five brains per sample.

Compared with the control group, the CUMS+D group showed significant increases in SOD (Figure 3A) and catalase (Figure 3B) activities, along with a significant decrease in GSH content (Figure 3C). CGA administration at the doses of 100 and 200 mg/kg significantly normalized the increase in SOD activity induced by CUMS+D treatment. A similar trend was observed for catalase activity following CGA treatment at all three doses (50, 100, and 200 mg/kg) in the CUMS+D group. However, 200 mg/kg CGA significantly increased catalase activity in the CGA control group. Moreover, CGA treatment at all the tested doses mitigated the reduction in GSH levels induced by CUMS+D exposure.

### 3.3. CGA Treatment Replenished NRF2 Protein Content and Normalized Keap1 mRNA Expression in the Brains of Fish Exposed to CUMS+D

**Figure 4:**
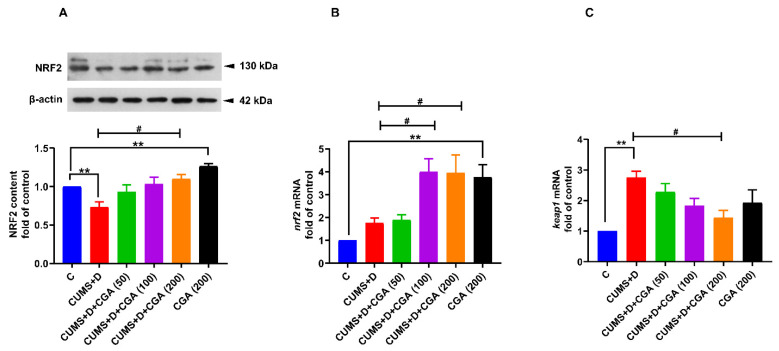
Effect of chlorogenic acid (CGA) treatment on nuclear factor erythroid 2-related factor 2 (NRF2) and kelch-like ECH-associated protein 1 (*keap1*) levels in the whole brains of chronic unpredictable mild stress and dextrose (CUMS+D)-exposed zebrafish. ((**A**), above) Representative Western blot image for NRF2 (original images are reported in Supplementary Material S2). ((**A**), below) ratio of NRF2 to β-actin (fold of control), and the mRNA expression levels of (**B**) *nrf2* and (**C**) *keap1*. A nonparametric test was used for the statistical analyses of *nrf2* and *keap1* mRNA levels (panels (**B**,**C**)). All original data are reported in Supplementary Material S3. Significant differences are indicated by ** *p* <0.01 when compared with the control group (**C**) and ^#^ *p* <0.05 compared with the CUMS+D group, analyzed by one-way ANOVA followed by Dunnett’s multiple comparisons test. Values represent the mean ± SEM (*n* = 3) from three independent samples, each consisting of a pool of five brains per sample.

NRF2 protein expression levels were significantly reduced in the whole brains of fish in the CUMS+D group (Figure 4A) when compared with those of fish in the control group. NRF2 content of the CUMS+D group demonstrated an increasing trend following CGA administration at all concentrations tested, reaching a significant change only at the maximum dose (200 mg/kg, Figure 4A). Additionally, whole brain NRF2 content was increased in the 200 mg/kg CGA control group when compared with that in the control group. The mRNA expression levels of *nrf2* (Figure 4B) appeared to increase non-significantly in the CUMS+D group compared with those in the control group; conversely, *keap1* mRNA expression levels (Figure 4C) were significantly elevated. CGA at doses of 100 and 200 mg/kg further increased *nrf2* mRNA levels in the treated groups when compared with those in the CUMS+D group. Moreover, CGA treatment led to a decrease in *keap1* gene expression levels at the 200 mg/kg dose. Additionally, administration of 200 mg/kg CGA alone significantly increased *nrf2* mRNA levels in the CGA control group when compared with those in the control group, whereas *keap1* mRNA levels were not significantly altered.

**Figure 5:**
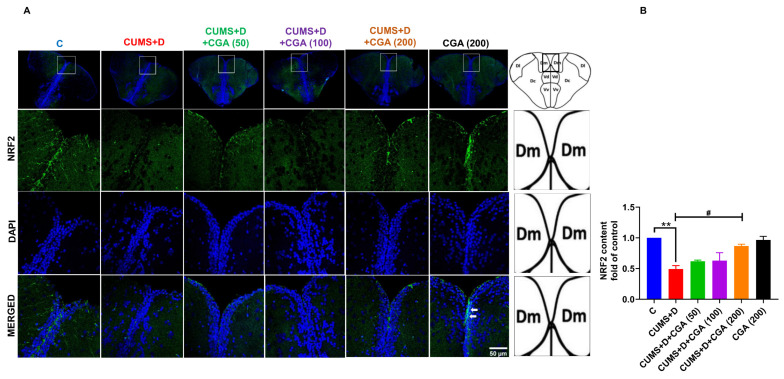
Effect of chlorogenic acid (CGA) treatment on nuclear factor erythroid 2-related factor 2 (NRF2) immunoreactivity in the telencephalon of chronic unpredictable mild stress and dextrose (CUMS+D)-exposed zebrafish. Representative images (**A**) showing immunostaining of NRF2 (green) counterstained with DAPI (blue), with white arrows indicating the colocalization of NRF2 with the nuclei, and (**B**) mean fluorescence intensity of NRF2 (depicted as fold of control). Scale bar: 50 µm. The images in the first row show: on the left, the entire telencephalon from animals belonging to the experimental groups (magnified 10×); on the right, a schematic graphical representation of telencephalon as the whole. The yellow boxes indicate the areas of interest, magnified 60× and analyzed in the subsequent images. The black box indicates the area of interest in the schematic graphical representation. The area surrounded by the boxes represent the medial zone of dorsal telencephalic area (Dm) and parts of the dorsal nucleus of ventral telencephalic area (Vd). All original data are reported in Supplementary Material S3. Significant differences are indicated by ** *p* < 0.01 when compared with the control group (C) and ^#^ *p* < 0.05 compared with the CUMS+D group, analyzed by one-way ANOVA followed by Dunnett’s multiple comparisons test. Values in B represent the mean ± SEM (*n* = 3) from three independent samples.

NRF2 immunostaining (Figure 5A,B) in the telencephalon of the brain revealed a significant decrease in NRF2 expression in the Dm (medial zone of the dorsal telencephalic area) and parts of the Vd (dorsal nucleus of the ventral telencephalic area) regions in the CUMS+D group when compared with that in the control group. Notably, the 200 mg/kg CGA dose prevented this reduction in NRF2 expression in CUMS+D-treated fish.

### 3.4. CGA Treatment Boosted the mRNA Expression Levels of NRF2-Target Antioxidant Genes in the Brains of Fish Exposed to CUMS+D

**Figure 6:**
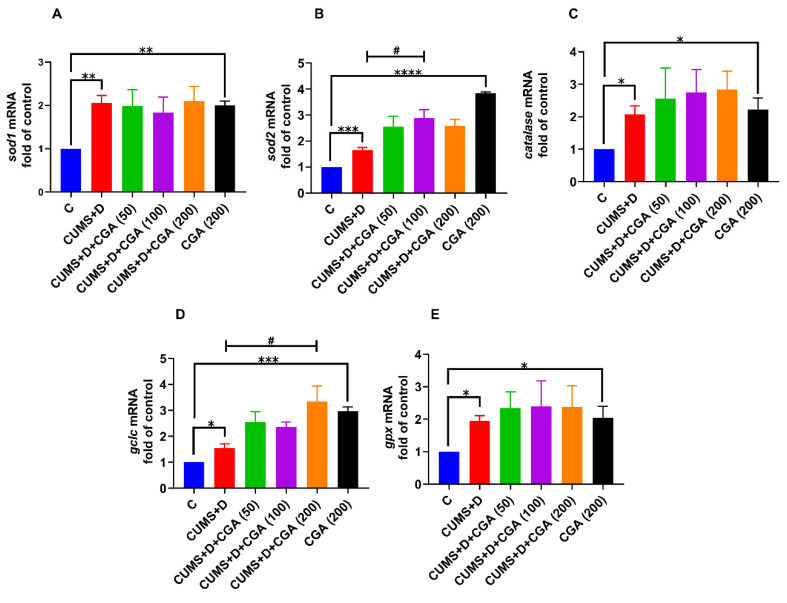
Effect of chlorogenic acid (CGA) treatment on the mRNA expression levels of nuclear factor erythroid 2-related factor 2 (NRF2)-target genes in the whole brains of chronic unpredictable mild stress and dextrose (CUMS+D)-exposed zebrafish. (**A**) *sod1*, (**B**) *sod2*, (**C**) *catalase*, (**D**) *gclc*, and (**E**) *gpx*. A nonparametric test was used for the statistical analyses of *sod1*, *sod2*, *catalase*, *gclc*, and *gpx* mRNA levels (panels (**A**), (**B**), (**C**), (**D**), and (**E**), respectively). All original data are reported in Supplementary Material S3. Significant differences are indicated by **** *p* < 0.0001, *** *p* < 0.001, ** *p* < 0.01, and * *p* < 0.05 when compared with the control group (C); and ^#^ *p* < 0.05 compared with the CUMS+D group, analyzed by one-way ANOVA followed by Dunnett’s multiple comparisons test. Values represent the mean ± SEM (*n* = 3) from three independent samples, each consisting of a pool of five brains per sample.

In the CUMS+D group, the mRNA levels of NRF2-target genes, *sod1* (Figure 6A), *sod2* (Figure 6B), *catalase* (Figure 6C), *gclc* (Figure 6D), and *gpx* (Figure 6E), were significantly increased compared with those in the control group. CGA administration further enhanced mRNA expression levels, specifically for *sod2* and *gclc* at the 100 and 200 mg/kg doses, respectively. The expression of *sod1* was unmodified by CGA treatment, whereas a trend toward increase was observed for *catalase* and *gpx* expression levels, which did not reach statistical significance. Notably, treatment with 200 mg/kg CGA alone markedly increased the mRNA levels of all five genes in the CGA control group when compared with those in the control group.

### 3.5. CGA Treatment Restored GFAP Protein Content in the Brains of Fish Exposed to CUMS+D

**Figure 7:**
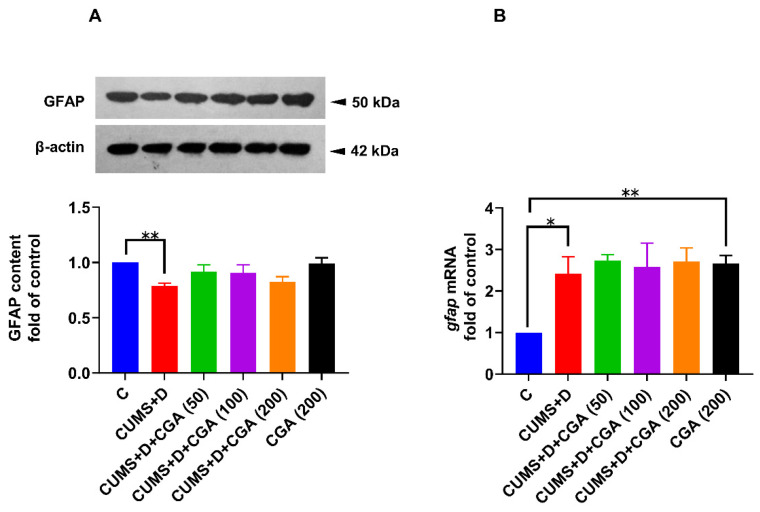
Effect of chlorogenic acid (CGA) treatment on glial fibrillary acidic protein (GFAP) expression in the whole brain of chronic unpredictable mild stress and dextrose (CUMS+D)-exposed zebrafish. ((**A**), above) Representative Western blot image for GFAP ((**A**), below), ratio of GFAP to β-actin (fold of control, original images in Supplementary Material S2), and (**B**) mRNA expression levels of *gfap*. A nonparametric test was used for the statistical analysis of the *gfap* mRNA content. All original data are reported in Supplementary Material S3. Significant differences are indicated by ** *p* < 0.01 and * *p* < 0.05 when compared with the control group (C), analyzed by one-way ANOVA followed by Dunnett’s multiple comparisons test. Values represent the mean ± SEM (*n* = 3) from three independent samples, each consisting of a pool of five brains per sample.

The CUMS+D group showed reduced GFAP protein levels in the whole brain compared with the control group (Figure 7A). CGA treatment at all tested doses did not affect GFAP protein content (Figure 7A). In the CGA control group treated with 200 mg/kg of CGA alone, GFAP protein content was not significantly different from that in the control group (Figure 7A). Additionally, the CUMS+D group showed increased *gfap* mRNA levels (Figure 7B); CGA-treated groups revealed no significant differences in *gfap* mRNA levels when compared with those in the CUMS+D group (Figure 7B). Interestingly, CGA at 200 mg/kg alone elevated *gfap* mRNA levels in the CGA control group when compared with those in the control group.

**Figure 8:**
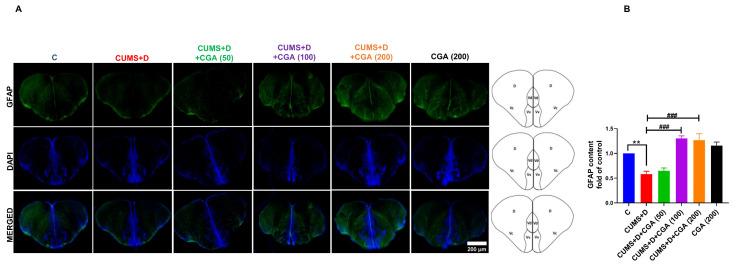
Effect of chlorogenic acid (CGA) treatment on glial fibrillary acidic protein (GFAP) immunoreactivity in the telencephalon of chronic unpredictable mild stress and dextrose (CUMS+D)-exposed zebrafish. (**A**) Representative images showing immunostaining of GFAP (green) counterstained with DAPI (blue), and (**B**) mean fluorescence intensity of GFAP (depicted as fold of control). Scale bar: 200 µm. All original data are reported in Supplementary Material S3. Significant differences are indicated by ** *p* < 0.01 when compared with the control group (C), and ^###^ *p* < 0.001 compared with the CUMS+D group, analyzed by one-way ANOVA followed by Dunnett’s multiple comparisons test. Values in B represent the mean ± SEM (*n* = 3) from three independent samples.

GFAP expression (Figure 8A,B) was lower in the telencephalon of the CUMS+D group when compared with that in the control group. CGA treatment significantly prevented this attenuation in GFAP expression, with both the 100 and 200 mg/kg doses resulting in significant remedial effects.

## 4. Discussion

In this study, we found that fasting blood glucose levels in zebrafish in the CUMS+D group were approximately triple those of their control-group cohorts, consistent with previous findings [8,28]. CGA, administered at a dose of 200 mg/kg, partially reversed this elevation. Significant reductions in glucose and insulin concentrations following acute CGA administration in clinical trials provide supporting evidence for its potential to improve glucose regulation [29]. The fast-acting hypoglycemic effects of CGA have been demonstrated in db/db mice, wherein a single intraperitoneal administration of 250 mg/kg CGA significantly reduced fasting blood sugar levels within 10 min, an effect that was found to be sustained for an additional 30 min after a glucose challenge, likely through AMPK activation-induced glucose transport in the skeletal muscle [30]. Studies performed in silico and in vitro have highlighted CGA’s inhibitory action on key enzymes involved in glucose metabolism, including glucose-6-phosphatase, α-amylase, and α-glucosidase [31,32]. This suggests a link between enzyme inhibition and reduced glucose uptake in the digestive tract, which may account for CGA’s hypoglycemic effects. CGA-containing extracts have been shown to reduce glucose transporter 2 (GLUT2) expression, thereby affecting glucose transport in intestinal epithelial cells [33], which may be responsible for the observed lowering of fasting blood glucose levels. In zebrafish larvae, acute CGA treatment produced protective effects against alloxan-induced pancreatic islet damage [34]. Given the similarity in glucose regulation pathways between zebrafish and humans [35], CGA may operate through similar mechanisms in both species.

Although no studies are available on the hypoglycemic properties of CGA in dextrose-induced hyperglycemic fish, a recent study documented that the oral administration of CGA-containing *Turnera subulata* Sm. flower extract in adult zebrafish counteracted the hyperglycemic effect of dextrose treatment [36]. Similarly, diet-induced metabolic disruptions, including elevated fasting blood glucose levels, were paralleled by disruptions in brain redox homeostasis, wherein CGA-containing *Antirhea borbonica* extract exhibited a preventative effect in adult zebrafish [37].

We explored CGA’s potential to counteract CUMS+D exposure-induced brain redox imbalance by measuring SOD and catalase enzyme activities and GSH content. CUMS+D exposure significantly increased SOD and catalase activities. These findings both corroborate and contradict previously published reports. While some studies have demonstrated elevated brain SOD and catalase activities in zebrafish and rodent models of hyperglycemia [38,39], others have observed the opposite [40]. Similarly, chronic stress paradigms in rodents have produced mixed results, with some reporting increases [41] and others decreases in antioxidant enzyme activities [42]. To date, no other study has investigated brain antioxidant enzyme activity following a 14-day CUMS regimen in zebrafish. Our previous work showed that CUMS resulted in significantly elevated SOD but not catalase activity, whereas CUMS+D treatment produced a further increase in SOD activity compared with CUMS-only exposure. Moreover, catalase activity elevation was observed only in the CUMS+D group [8]. In contrast, seven days of chronic stress exposure led to reductions in both SOD and catalase activities, accompanied by increased ROS production in another zebrafish study [43]. These inconsistencies suggest that the type and duration of the stress protocol may critically influence oxidative stress markers, at least in the context of chronic stress. Notably, GSH levels were found to be decreased after CUMS+D exposure in the present study, aligning with previous reports of reduced GSH in the brains of diabetic rodents and fish [36,44], and chronically stressed rodents and fish [42,45]. Our previous study found no additive or synergistic effects of combined stress and hyperglycemia, as both conditions in isolation and in combination reduced GSH content [8]. Together, these results suggest that the observed increases in SOD and catalase activities, along with reduced GSH, may represent compensatory responses to oxidative imbalance induced by CUMS+D exposure. It is also important to note that elevated ROS levels, a direct measure of oxidative stress, remain a consistent outcome in both chronic stress and hyperglycemia. Future studies should additionally include assessments of the activities of other antioxidant enzymes, such as GPX, glutathione reductase, and glutathione S-transferase, to further substantiate this hypothesis. We noted that all three CGA doses differentially corrected these alterations, with the 100 mg/kg dose showing consistent improvements, supporting CGA’s effectiveness in counteracting redox imbalance. Similarly, acute CGA treatment, administered intraperitoneally at 0- and 2 h post-ischemic injury in a rat model, protected against oxidative damage and blood–brain barrier injury [46]. Other reports of therapeutic effects following acute CGA therapy include the restoration of GSH levels and normalization of SOD, catalase, and GPX enzyme activities in gastric tissue, as demonstrated in an in vivo study performed in mice [47]. Another study highlighted the antioxidative and anti-inflammatory effects of a single intraperitoneal injection of CGA in rats with potassium dichromate-induced acute hepato-nephrotoxicity [48]. Previous studies on CGA’s antioxidant effects in the brain have demonstrated its ability to replenish GSH and boost antioxidant enzyme activities [49,50]. These studies reported decreased SOD and catalase activities, which were subsequently reversed by CGA treatment, a result not observed in our study. The discrepancies may be attributed to differences in experimental conditions, as these studies were not specifically focused on hyperglycemia. Given the limited research on CGA’s anti-diabetic effects specific to brain tissue, a conclusive interpretation remains elusive. However, the normalization of SOD and catalase activities observed in our study may be attributed to CGA’s direct scavenging of superoxide and hydroxyl radicals [10], thereby reducing the oxidative burden on these enzymes.

Redox imbalance is linked to NRF2 pathway dysregulation in the diabetic brain [51]; however, evidence is scarce when it comes to diabetes distress. We measured NRF2 protein and gene expression levels, as well as *keap1* gene expression levels. In the CUMS+D group, NRF2 protein levels were significantly reduced, whereas *nrf2* gene expression was elevated, likely as a compensatory response, consistent with studies on diabetic rodents [52,53] and hyperglycemic zebrafish [38]. Additionally, *keap1* expression was elevated, aligning with studies showing reduced NRF2 protein content and enhanced KEAP1 expression in diabetic rodents [54]. One mechanism through which CGA provides neuroprotection is by enhancing NRF2 expression and facilitating its nuclear translocation [55]. Our observations corroborate these findings, as CGA enhanced NRF2 protein and mRNA expressions and simultaneously reduced *keap1* mRNA expression, particularly at the 200 mg/kg dose. Studies suggest that CGA’s NRF2-promoting activity primarily depends on its ability to suppress KEAP1 at both transcriptional [56] and translational [57] levels, as well as the inhibition of KEAP1-NRF2 binding [58]. Immunostaining revealed decreased NRF2 expression in the Dm and parts of the Vd areas of the telencephalon after CUMS+D exposure, which was restored by 100 and 200 mg/kg of CGA. The zebrafish telencephalon is believed to be functionally and evolutionarily similar to the mammalian telencephalon, with the Dm and Vd areas hypothesized to be analogous to the mammalian amygdala [59] and basal ganglia [60], respectively. To the best of our knowledge, this is the first study to investigate CGA’s neuroprotective potential against diabetes distress in these specific brain regions of the zebrafish telencephalon.

In this study, CUMS+D treatment significantly impacted brain oxidative stress markers. To correlate these changes with the mRNA levels of antioxidant genes, we assessed *sod1*, *sod2*, *catalase*, *gclc*, and *gpx* levels. CUMS+D exposure elevated the mRNA levels of these genes, indicating redox imbalance, which is consistent with existing studies [38,61]. CGA administration further enhanced *sod2* and *gclc* expressions at 100 and 200 mg/kg doses, respectively. The increase in *gclc*, a key enzyme in GSH synthesis, may compensate for CUMS+D-induced GSH reduction. Supporting evidence for CGA-induced increases in the gene expressions of *sod1*, *sod2*, and *gpx* in the liver under diabetic conditions can be traced to rodent studies [62]. However, data on CGA’s efficacy in enhancing these genes in the brain under hyperglycemia are lacking, indicating a need for further research. We hypothesize that CUMS+D treatment induces stress on the NRF2 pathway, resulting in NRF2 protein reduction. CGA treatment, especially at 200 mg/kg, appears to reverse this effect, demonstrating neuroprotective action in the adult zebrafish brain. The increase in the mRNA expression of NRF2-target genes despite its reduction may be similar to the compensatory increase in *nrf2* mRNA expression. Western blotting analyses have revealed reductions in protein levels of NRF2-target genes, particularly GPX4 and heme oxygenase-1, in the brains of both diabetic and chronically stressed rats [63,64]. The possibility of protein-level reductions in other antioxidant enzymes, such as SOD1, SOD2, and catalase, under diabetes distress remains to be explored and constitutes a major limitation of our study. Another key point to consider is that transcription factors other than NRF2 can also induce the transcription of antioxidant genes in response to oxidative stress, most notably, members of the forkhead box class O (FOXO) family. FOXO activation may occur as a compensatory mechanism in response to reduced NRF2, potentially enhancing the transcription of antioxidant genes. Notably, both NRF2 and FOXO are known to be protective against oxidative stress and share similar target antioxidant genes, including catalase and SOD2 [65]. Discrepancies between mRNA and protein expression levels are frequently observed in molecular studies and can arise from several mechanisms; these include post-transcriptional modifications affecting mRNA stability and translational efficiency, and post-translational modifications influencing protein stability, half-life, or interactions. Furthermore, under conditions of elevated oxidative stress, NRF2 may bind to the promoter region of its own gene to enhance its transcription, establishing a positive autoregulatory feedback loop [66]. The potential for such NRF2 self-regulation could be assessed in future studies using chromatin immunoprecipitation assays; however, whether this mechanism operates under conditions characterized by overall reduction in NRF2 protein remains uncertain.

Accumulating evidence shows that physiological stress leads to morphological alterations in glial cells, accompanied by impaired cerebral homeostasis. Zebrafish radial glial cells, which are largely proliferative and persist in neurogenic niches [67], are speculated to be comparable to the mammalian astroglia, as both populations express similar molecular markers, including GFAP, and share morphological attributes, thereby potentially contributing to brain homeostasis via shared mechanisms [68]. We measured GFAP levels in the whole brain using immunoblotting and performed immunofluorescence staining to label glia in the telencephalon. CUMS+D treatment reduced whole brain GFAP protein levels in diseased fish when compared with those in the control group. Studies conducted using rodent models have revealed GFAP protein levels to be reduced following CUMS exposure [69], whereas hyperglycemia has been associated with elevated GFAP expression [70]. Our findings suggest that the effect of CUMS may override that of hyperglycemia, resulting in an overall decrease in GFAP protein content. This interpretation is supported by evidence that astroglial response in disease pathophysiology is context-specific, influenced by several factors such as disorder type, stage, and duration, as well as the presence of comorbidities [13]. It is crucial to mention that although GFAP is a widely used molecular marker for reactive astroglia, further investigations should incorporate additional astroglial-specific markers beyond GFAP to obtain a more comprehensive understanding of astroglial responses in diabetes distress. For instance, chronic stress exposure in rodents has been associated with reductions in the expression of several astroglial markers alongside GFAP, including S100 calcium-binding protein B (S-100β) [71], glutamate transporter-1 (GLT-1) [72], excitatory amino acid transporter-2/glutamate–aspartate transporter (GLAST) [73], and aquaporin-4 [74]. In diabetic models, rats have been reported to exhibit increased protein expression of both GFAP and S-100β [75]; however, reduced GLT-1 expression was associated with an increased risk of postoperative cognitive impairment in diabetic mice [76]. Similarly, hyperglycemic mice have demonstrated reduced hippocampal expression of GLAST and GLT-1 [77]. These findings suggest that impaired astroglial glutamate uptake is a shared pathological feature of both hyperglycemia and chronic stress. Indeed, reduced glutamate clearance by astrocytes is strongly associated with oxidative stress and neurotoxicity in the CNS, indicating that it is a common outcome of both hyperglycemic and chronic stress conditions [78]. In the present study, CGA treatment appeared to reverse the reduction in GFAP protein expression, exhibiting a neuroprotective effect. A notable finding in our study was that the CUMS+D group displayed increased brain *gfap* mRNA levels, consistent with previous findings in hyperglycemic fish [79]. In contrast, another zebrafish study revealed no changes in brain *gfap* mRNA levels even after “prolonged unpredictable strong chronic stress” lasting 5 weeks [80]. As no prior study has evaluated *gfap* mRNA levels after combined CUMS+D exposure, we can only speculate that this increase indicates a compensatory response to diabetes distress or reduced GFAP protein expression. GFAP expression in the telencephalon was significantly reduced in the CUMS+D group when compared with that in the control group, further suggesting that the detrimental effects of CUMS exposure on astroglial health in the zebrafish brain predominates over those of hyperglycemia. Rodent models of chronic stress show similar findings, including attenuated GFAP immunostaining, decreased population of GFAP-positive cells [81], and astroglial atrophy [82]. Conversely, diabetic rats exhibit increased GFAP immunostaining in key brain regions, such as the cerebellar cortex, which has been associated with reactive astrogliosis [83]. CGA treatment significantly restored GFAP expression in the telencephalon of diseased fish, highlighting its neuroprotective action. To our knowledge, this is the first study to investigate CGA’s potential remedial effects against diabetes distress-associated glial dysfunction. Although a precise explanation is difficult to formulate given the scarcity of studies investigating the involvement of zebrafish glial cells in hyperglycemia, our findings suggest that the combined insult of chronic stress and hyperglycemia is detrimental to radial glial morphology. Therefore, DD may adversely affect astroglia; whether dysfunctional astroglia precede other homeostatic disruptions or result as collateral damage, remains unclear. Given that healthy astroglia contribute to neuroprotection through the release of antioxidants, including GSH [78], we speculate that CGA helps maintain astroglial redox homeostasis. NRF2 overexpression in astroglia has been demonstrated to protect against neurodegeneration [84], with GSH production playing a key role in mediating this neuroprotective action [85]. Moreover, in a mouse model of neurodegeneration, CGA showed therapeutic potential by boosting antioxidant activity in astroglia [86]. Our findings suggest that astroglial cells are valuable targets in comorbidities, and chronic stress may exacerbate compromised brain homeostasis by endangering astroglial health.

## 5. Conclusions

In summary, CUMS+D exposure imposed significant stress on the NRF2 signaling pathway and negatively impacted the radial glial population in the brain, especially in the telencephalon. CGA treatment, especially at the 200 mg/kg dose, largely alleviated these effects and normalized fasting blood glucose levels. Our study provides novel molecular insights into the neurological aspects of diabetes distress; however, it is not without its limitations. We examined the effect of acute doses of CGA rather than a chronic treatment regimen, primarily because of the unique experimental design of our study, which involved prior exposure to CUMS+D insults, resulting in low survivability of zebrafish following repeated intraperitoneal injections. This limitation prevented us from conducting longitudinal studies that could have helped reveal the long-term effects of CGA treatment and its implications for disease progression. Nonetheless, our findings highlight the short-term therapeutic effects of acute CGA administration, suggesting that CGA holds therapeutic potential for modulating redox signaling in hyperglycemia-associated CNS injury, highlighting the benefits of targeting antioxidant pathways for disease management. Chronic treatment with CGA may yield better therapeutic outcomes by facilitating the elucidation of the temporal pattern of disease marker expression, thus identifying critical time points for precise intervention and disease management. Targeting NRF2 in disease states appears to be both time- and expression-dependent, emphasizing the need for longitudinal studies to determine the precise timing, duration, and extent of NRF2 modulation required for optimal therapeutic benefit. Future research should focus on validating and extending these findings in rodent models to further elucidate the underlying mechanistic pathways and their potential crosstalk with other regulatory networks. In parallel, investigating the long-term effects of CGA on vital organs such as the pancreas, liver, and skeletal muscle will be critical to understanding broader implications for disease progression and metabolic health. Another limitation of this study is that it did not include a comparative analysis of CGA with standard antidiabetic drugs, which would have provided a clearer understanding of its relative efficacy and translational potential. There remains considerable scope for future comparative efficacy studies involving both established and emerging therapeutic agents, particularly CGA, to better evaluate their clinical relevance in modulating NRF2 activity and related cellular defense mechanisms. In conclusion, the NRF2 antioxidative defense system is a promising target for drug development for both diabetes-related distress as well as other neurological disorders associated with metabolic dysfunction.

## Figures and Tables

**Table 1:** Primers used for qRT-PCR assays.

	Forward Primer (5′–3′)	Reverse Primer (5′–3′)	Accession ID
*nrf2*	AACGAGTTCTCCCTTCAGCA	ATTTTGTCGCCGATTTTGTC	BC152659
*keap1*	TGGATAACTACCTCTATGCCGT	CCTTGGTTAAATCCACCTAACAC	NM_182864
*sod1*	CAATGCTAACTTTGTCAGGCCA	CCTTCCCCAAGTCATCCTCC	BC165134
*sod2*	CTTGGGATAGATGTCTGGG	GTGGTCTGATTAATTGTGCG	XM_057353025
*catalase*	AACCAACAACCCTCCAGACAG	TCCGCTCTCGGTCAAAATGG	NM_130912
*gpx*	AGATGTCATTCCTGCACACG	AAGGAGAAGCTTCCTCAGCC	AY216589
*gclc*	AACCGACACCCAAAGATTCAGCACT	CCATCATCCTCTGGAAACACCTCC	XM_031693913
*gfap*	GGATGCAGCCAATCGTAAT	TTCCAGGTCACAGGTCAG	AH012040
*β-actin*	CGAGCAGGAGATGGGAACC	CAACGGAAACGCTCATTGC	BC045846

## Data Availability

This article and its supplementary files include all data generated or analyzed during this study. All original data for this study are included in the Supplementary Materials S3. Additional inquiries may be addressed to the corresponding authors.

## Supplementary Materials

The following supporting information can be downloaded at: https://www.mdpi.com/article/10.3390/antiox14121386/s1, S1: Survival analysis after chlorogenic acid (CGA) administration; S2: Original blot images; S3: Open Data.

## Abbreviations

The following abbreviations are used in this manuscript:

C	Control group
CGA	Chlorogenic acid
CNS	Central nervous system
CUMS	Chronic unpredictable mild stress
D	Dextrose
DD	Diabetes distress
GCLC	Glutamate-cysteine ligase catalytic subunit
GFAP	Glial fibrillary acidic protein
GLAST	Glutamate–aspartate transporter
GLT-1	Glutamate transporter-1
GPX	Glutathione peroxidase
GSH	Glutathione
KEAP	Kelch-like ECH-associated protein 1
NRF2	Nuclear factor erythroid 2-related factor 2
S-100β	S100 calcium-binding protein B
SOD	Superoxide dismutase
