# Supplementary material for: Chlorogenic Acid Alleviates the Detrimental Effects of Concurrent Hyperglycemia and Chronic Stress on Brain Homeostasis by Modulating Antioxidative Defense in Adult Zebrafish
Source: Antioxidants (Basel). 2025 Nov 21;14(12):1386. doi: 10.3390/antiox14121386 (PMC12730118; doi:10.3390/antiox14121386)
Supplement: Supplementary file 1 [file antioxidants-14-01386-s001.zip › antioxidants-3939156-supplementary.pdf]

SUPPLEMENTARY MATERIAL

Survival analysis after chlorogenic acid (CGA) administration S1

A dose-response trial was conducted to determine the optimal dose and duration of CGA to be administered to the fish across different experimental groups. Fish were intraperitoneally injected with CGA at doses of 50, 100, 200, and 400 mg/kg of body weight. The 400 mg/kg dose resulted in only 50% survival 24 hours post-injection (**figure S1.a**), indicating toxicity at this dose. In contrast, fish that received CGA at doses of 50, 100, and 200 mg/kg exhibited over 80% survival 24 hours post-injection. Consequently, these three lower doses were selected for the full-fledged experiments as they were well-tolerated.

Upon completion of the 14-day CUMS+Dextrose treatment, fish in their respective experimental groups received CGA at the doses of 50, 100, and 200 mg/kg. Survival was monitored from Day 1 to Day 17 during consecutive CGA intraperitoneal injections. CUMS+Dextrose exposure occurred from Days 1 to 14, with CGA administered at 24-hour intervals on Days 15 and 16. Similarly, a single CGA intraperitoneal injection was administered on Day 15 following 14 days of CUMS+Dextrose exposure, with survival monitored from Day 1 to Day 16. Survival was estimated using the Kaplan-Meier method. Endpoints in this study were defined as achieving over 70% survival 24 hours post-CGA administration. The survival curve was plotted with time lapse (in days) as a function of percent survival. Survival curves were compared using the log-rank (Mantel-Cox) test.

A second intraperitoneal injection, given 24 hours after the first, resulted in more than 50% mortality across all the experimental groups (**figure S1.b**). Therefore, only a single, acute intraperitoneal dose of CGA was selected for the study, which resulted in over 70% survival by the end of the treatment (**figure S1.c**). The survival curves of the experimental groups did not show significant differences in both instances. It is possible that prior exposure to significant physiological stress from combined hyperglycemia and CUMS rendered the animals more vulnerable to the effects of repeated intraperitoneal injections. Due to high mortality with consecutive CGA injections, acute CGA doses

were used in the final study. Consequently, the protocol was adjusted to include 14 days of CUMS+Dextrose treatment followed by a single CGA injection on Day 15.

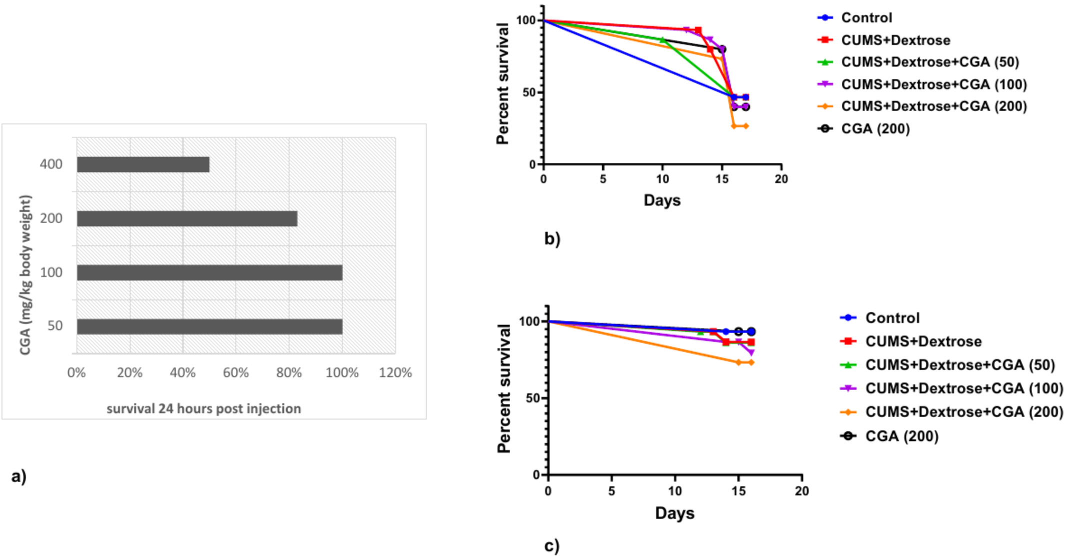

Supplementary figure S1: **a)** Dose-response study of survival of adult zebrafish 24 hours post intraperitoneal injections of chlorogenic acid (CGA) at the doses of 50, 100, 200, and 400 mg/kg per of body weight. Survival analysis of adult zebrafish after CUMS+Dextrose exposure followed by CGA treatment: **b)** consecutive intraperitoneal injections of CGA on the 15th and 16th days and **c)** a single intraperitoneal injection of CGA on the 15th day. Data was obtained from n=15 fish.

WESTERN BLOTS (ORIGINAL BLOT IMAGES) S2

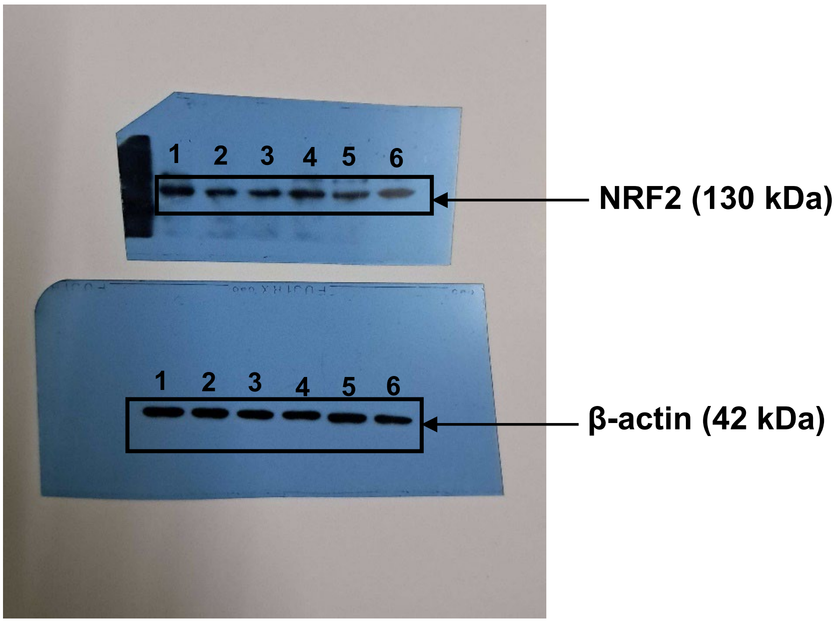

Supplementary figure S2.a: Full scanned blots of NRF2 and β-actin used in **Fig. 4. A. Lane 1**: Control. **Lane 2**: CUMS+Dextrose. **Lane 3**: CUMS+Dextrose+CGA (50). **Lane 4**: CUMS+Dextrose+CGA (100). **Lane 5:** CUMS+Dextrose+CGA (200). **Lane 6:** CGA (200).

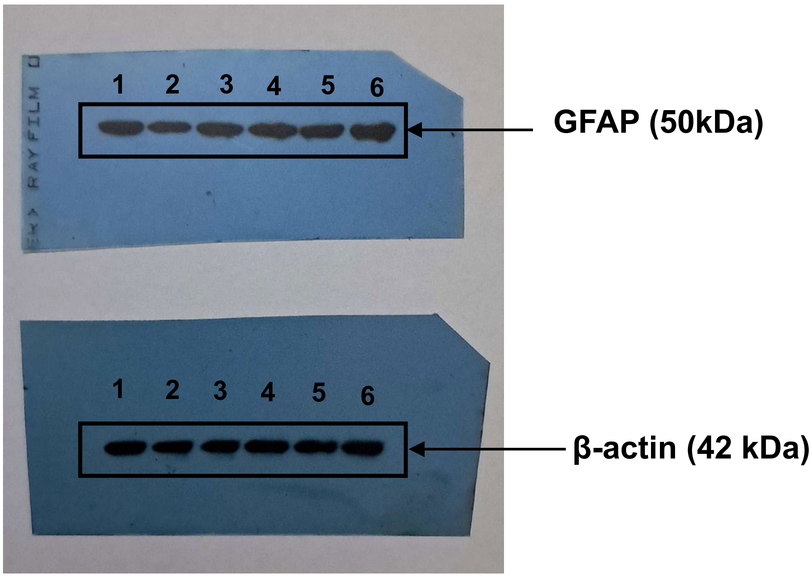

Supplementary figure S2.b: Full scanned blots of GFAP and β-actin used in **Fig. 7. A. Lane 1**: Control. **Lane 2**: CUMS+Dextrose. **Lane 3**: CUMS+Dextrose+CGA (50). **Lane 4**: CUMS+Dextrose+CGA (100). **Lane 5:** CUMS+Dextrose+CGA (200). **Lane 6:** CGA (200).

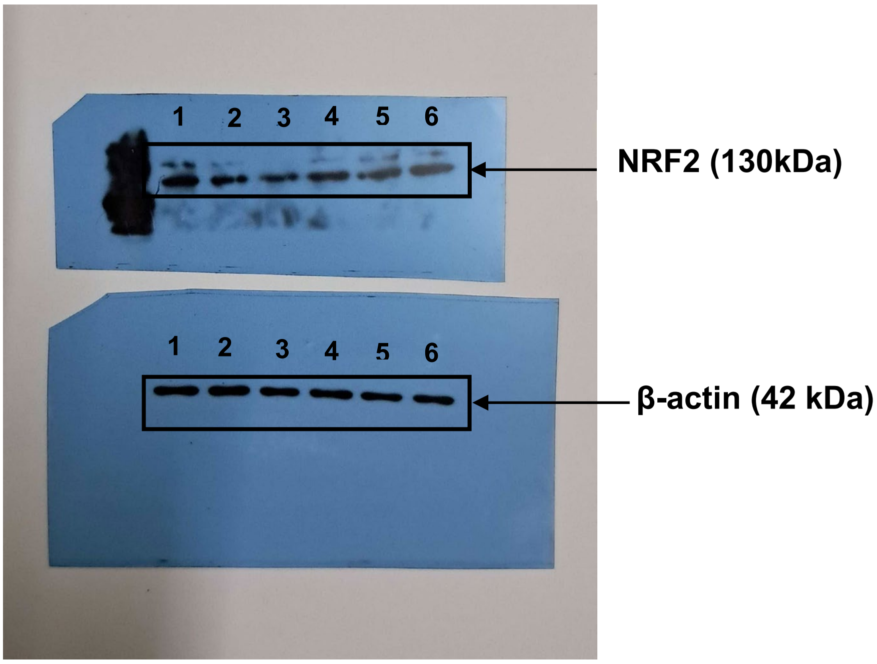

Supplementary figure S2.c: Full scanned blots of NRF2 and β-actin. **Lane 1**: Control. **Lane 2**: CUMS+Dextrose. **Lane 3**: CUMS+Dextrose+CGA (50). **Lane 4**: CUMS+Dextrose+CGA (100). **Lane 5:** CUMS+Dextrose+CGA (200). **Lane 6:** CGA (200). Biological replicate for figure 4.

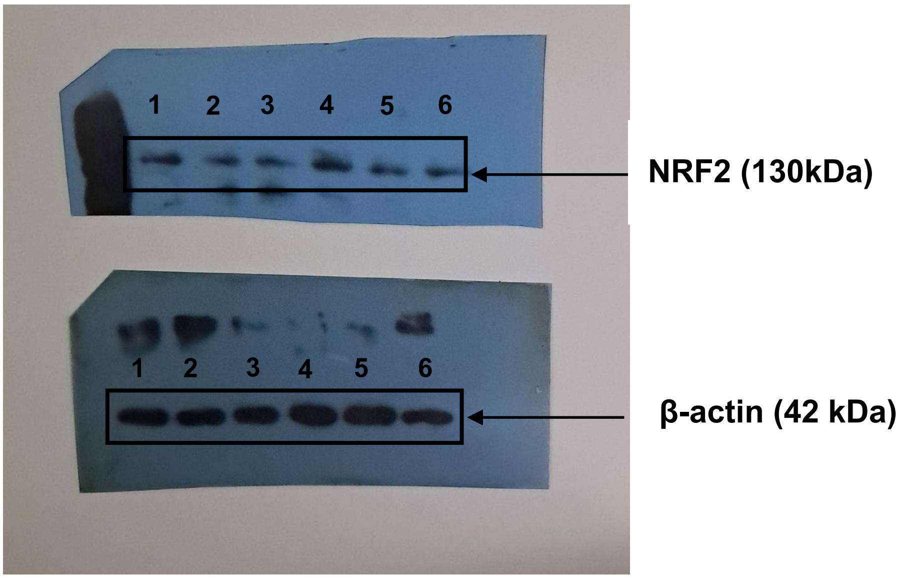

Supplementary figure S2.d: Full scanned blots of NRF2 and β-actin. **Lane 1**: Control. **Lane 2**: CUMS+Dextrose. **Lane 3**: CUMS+Dextrose+CGA (50). **Lane 4**: CUMS+Dextrose+CGA (100). **Lane 5:** CUMS+Dextrose+CGA (200). **Lane 6:** CGA (200). Biological replicate for figure 4.

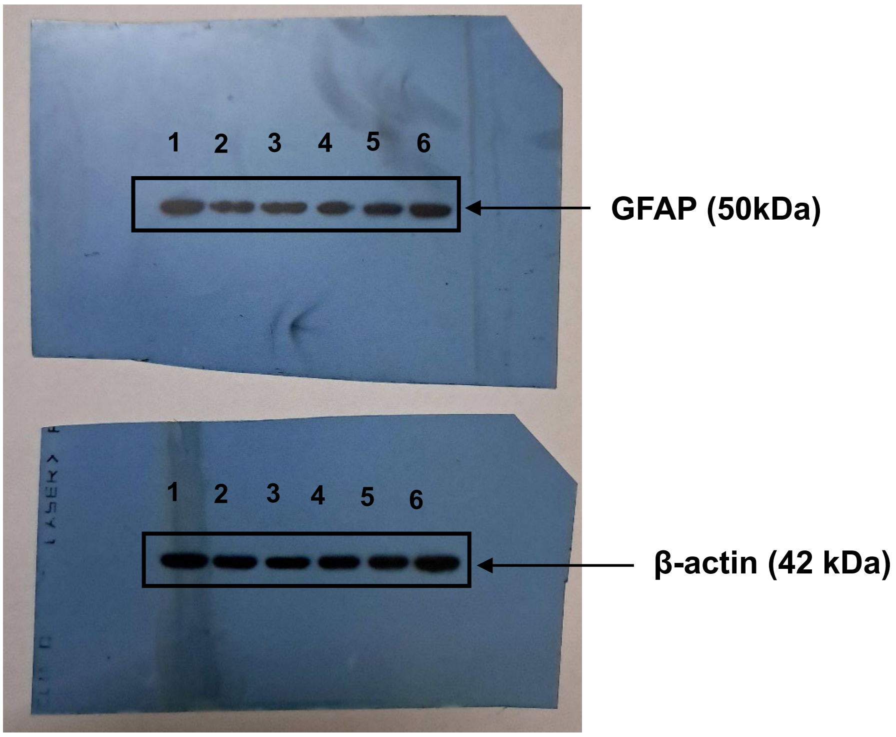

Supplementary figure S2.e: Full scanned blots of GFAP and β-actin. **Lane 1**: Control. **Lane 2**: CUMS+Dextrose. **Lane 3**: CUMS+Dextrose+CGA (50). **Lane 4**: CUMS+Dextrose+CGA (100). **Lane 5:** CUMS+Dextrose+CGA (200). **Lane 6:** CGA (200). Biological replicate for figure 7.

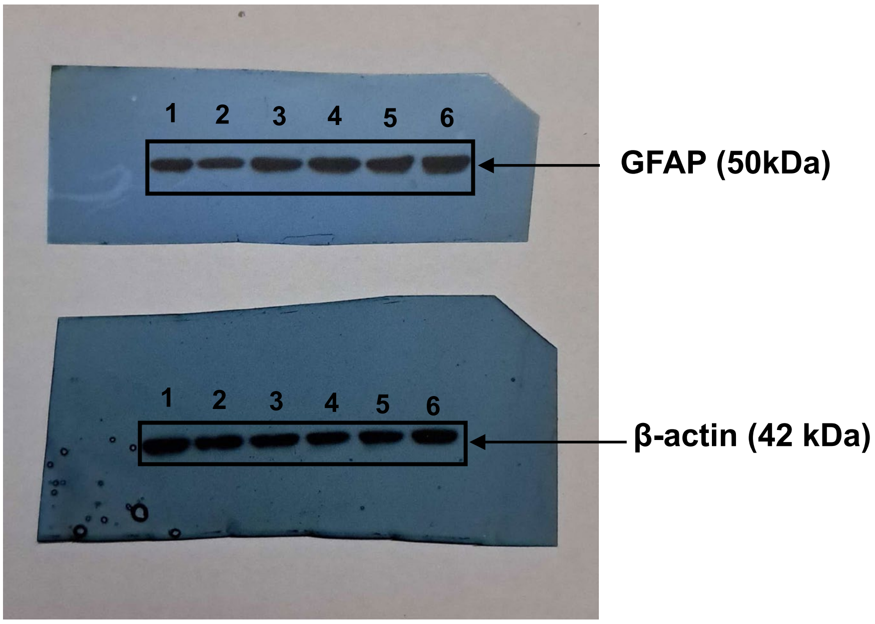

Supplementary figure S2.f: Full scanned blots of GFAP and β-actin. **Lane 1**: Control. **Lane 2**: CUMS+Dextrose. **Lane 3**: CUMS+Dextrose+CGA (50). **Lane 4**: CUMS+Dextrose+CGA (100). **Lane 5:** CUMS+Dextrose+CGA (200). **Lane 6:** CGA (200). Biological replicate for figure 7.

OPEN DATA S3

Fasting blood glucose levels	C	CUMS+D	CUMS+D+CGA (50)	CUMS+D+CGA (100)	CUMS+D+CGA (200)	CGA (200)
	54	167	109	136	68	50
	57	142	119	164	114	69
	81	148	185	95	58	53
	56	172	151	80	87	68
	65	155	146	199	160	72
	48	188	169	128	57	69
	46	177	138	174	103	57
	67	147	113	175	72	58
	61	166	143	192	72	70
	54	175	189	100	68	44
	61	156	97	181	77	74
	68	167	173	90	109	63
	82	167	122	194	178	39
	77	171	147	125	189	80
	59	145	144	175	77	50

Table S1.a: Fasting blood glucose levels (figure 2 of the manuscript). Control (C), CUMS+Dextrose (CUMS+D), CUMS+Dextrose+CGA (CUMS+D+CGA 50, 100 or 200 mg/kg), and CGA (200 mg/kg).

(A) SOD	C	CUMS+D	CUMS+D+CGA (50)	CUMS+D+CGA (100)	CUMS+D+CGA (200)	CGA (200)
	1.250972	3.084373	2.241967	1.796032	1.75558	1.920897
	1.585051	2.421069	2.258343	1.221155	1.437072	1.74497
	1.520885	2.303151	2.699161	1.470575	1.211131	1.601843
(B) Catalase	C	CUMS+D	CUMS+D+CGA (50)	CUMS+D+CGA (100)	CUMS+D+CGA (200)	CGA (200)
	7.691777498	11.0623475	9.24823942	8.083826693	7.633338962	12.59597203
	8.206805	11.91415	9.654582	8.408435	7.686827	12.11868
	8.063852	12.12993	9.238962	7.911405	8.014188	13.20672
(C) GSH	C	CUMS+D	CUMS+D+CGA (50)	CUMS+D+CGA (100)	CUMS+D+CGA (200)	CGA (200)
	3.201287	1.075368	1.778493	2.729779	2.018382	2.688419
	2.895221	1.141544	1.6875	3.226103	1.64614	2.53125
	3.110294	1.125	2.01011	2.274816	1.497243	2.481618

Table S1.b: (A) SOD activity; (B) Catalase activity; (C) GSH levels Fasting blood glucose levels (figure 3 of the manuscript). Control (C), CUMS+Dextrose (CUMS+D), CUMS+Dextrose+CGA (CUMS+D+CGA 50, 100 or 200 mg/kg), and CGA (200 mg/kg).

(A) NRF2	C	CUMS+D	CUMS+D+CGA (50)	CUMS+D+CGA (100)	CUMS+D+CGA (200)	CGA (200)
	1	0.8	1.1	1.2	1.0	1.3
	1	0.6	0.8	0.9	1.2	1.3
	1	0.8	0.9	1.0	1.1	1.2
(B) *nrf2* mRNA	C	CUMS+D	CUMS+D+CGA (50)	CUMS+D+CGA (100)	CUMS+D+CGA (200)	CGA (200)
	1	1.684671	2.29278	4.210343	5.029754	4.678509
	1	1.427715	1.868248	4.852839	4.391322	3.846192
	1	2.166657	1.535351	2.960701	2.433802	2.754005
(C) *keap1* mRNA	C	CUMS+D	CUMS+D+CGA (50)	CUMS+D+CGA (100)	CUMS+D+CGA (200)	CGA (200)
	1	3.105808	2.22819	2.209022	1.518849	2.584012
	1	2.414015	1.832192	1.892122	1.805843	2.05737
	1	2.747425	2.786222	1.399586	1.000000	1.148698

Table S1.c: (A) ratio of NRF2 to β-actin (fold of control); (B) *nrf2* mRNA expression; (C) *keap1* mRNA expression (figure 4 of the manuscript). Control (C), CUMS+Dextrose (CUMS+D), CUMS+Dextrose+CGA (CUMS+D+CGA 50, 100 or 200 mg/kg), and CGA (200 mg/kg).

(B) NRF2 Content	C	CUMS+D	CUMS+D+CGA (50)	CUMS+D+CGA (100)	CUMS+D+CGA (200)	CGA (200)
	1	0.537358	0.61674	0.513891	0.918267	0.850133
	1	0.555583	0.652034	0.883001	0.866387	1.000000
	1	0.386503	0.589696	0.496711	0.821273	1.046788

Table S1.d: (B) NRF2 immunostaining quantification (figure 5 of the manuscript). Control (C), CUMS+Dextrose (CUMS+D), CUMS+Dextrose+CGA (CUMS+D+CGA 50, 100 or 200 mg/kg), and CGA (200 mg/kg).

(A) *sod1* mRNA	C	CUMS+D	CUMS+D+CGA (50)	CUMS+D+CGA (100)	CUMS+D+CGA (200)	CGA (200)
	1	2.406102	2.541021	2.540302	2.779836	2.143547
	1	1.853721	2.14459	1.376071	1.771535	1.796265
	1	1.911891	1.275326	1.583158	1.745759	2.049126
(B) *sod2* mRNA	C	CUMS+D	CUMS+D+CGA (50)	CUMS+D+CGA (100)	CUMS+D+CGA (200)	CGA (200)
	1	1.456226	3.126572	2.954226	3.050721	3.950988
	1	1.751666	1.757843	2.288535	2.50115	3.751992
	1	1.749262	2.753632	3.396526	2.190634	3.799648
(C) *catalase* mRNA	C	CUMS+D	CUMS+D+CGA (50)	CUMS+D+CGA (100)	CUMS+D+CGA (200)	CGA (200)
	1	2.285771	1.10637	1.872158	2.2965	2.580466
	1	2.383248	4.326962	2.256221	2.238543	1.490503
	1	1.530503	2.234824	4.129755	3.97451	2.581701
(D) *gclc* mRNA	C	CUMS+D	CUMS+D+CGA (50)	CUMS+D+CGA (100)	CUMS+D+CGA (200)	CGA (200)
	1	1.774279	1.968584	1.974486	2.76255	2.639065
	1	1.646439	2.357882	2.518582	2.760177	3.05656
	1	1.229603	3.32196	2.571913	4.524899	3.202617
(E) *gpx* mRNA	C	CUMS+D	CUMS+D+CGA (50)	CUMS+D+CGA (100)	CUMS+D+CGA (200)	CGA (200)
	1	1.836487	2.109395	2.295729	2.188798	1.89813
	1	2.258593	1.613322	1.090534	1.375575	1.490116
	1	1.772397	3.295297	3.79735	3.5801	2.722385

Table **S1.e**: (A) *sod1* mRNA expression; (B) *sod2* mRNA expression; (C) *catalase* mRNA expression; (D) gclc mRNA expression; (E) gpx mRNA expression (figure 6 of the manuscript). Control (C), CUMS+Dextrose (CUMS+D), CUMS+Dextrose+CGA (CUMS+D+CGA 50, 100 or 200 mg/kg), and CGA (200 mg/kg).

.

(A) GFAP	C	CUMS+D	CUMS+D+CGA (50)	CUMS+D+CGA (100)	CUMS+D+CGA (200)	CGA (200)
	1.0000	0.8108	1.0203	0.9508	0.8260	0.9965
	1.0000	0.8142	0.9242	0.7714	0.7472	0.8927
	1.0000	0.7446	0.8106	1.0039	0.9048	1.0792
(B) *gfap* mRNA	C	CUMS+D	CUMS+D+CGA (50)	CUMS+D+CGA (100)	CUMS+D+CGA (200)	CGA (200)
	1	3.171156	3.010783	3.305801	3.260309	4.392371
	1	2.305373	2.61172	2.979355	2.741566	2.273634
	1	1.79005	2.585118	1.469169	2.12874	2.928171

Table **S1.f**: (A) ratio of GFAP to β-actin (fold of control); (B) *gfap* mRNA expression (figure 7 of the manuscript). Control (C), CUMS+Dextrose (CUMS+D), CUMS+Dextrose+CGA (CUMS+D+CGA 50, 100 or 200 mg/kg), and CGA (200 mg/kg).

(B) GFAP	C	CUMS+D	CUMS+D+CGA (50)	CUMS+D+CGA (100)	CUMS+D+CG A (200)	CGA (200)
	1	0.520994	0.557674	1.234194	1.212838	1.272925
	1	0.697224	0.750262	1.404138	1.515191	1.174961
	1	0.528568	0.641418	1.268848	1.068634	1.024893

Table S1.g: (B) GFAP immunostaining quantification (fold of control) (figure 8 of the manuscript). Control (C), CUMS+Dextrose (CUMS+D), CUMS+Dextrose+CGA (CUMS+D+CGA 50, 100 or 200 mg/kg), and CGA (200 mg/kg).
